# Toxic Effects of the Mixture of Phthalates and Bisphenol A—Subacute Oral Toxicity Study in Wistar Rats

**DOI:** 10.3390/ijerph17030746

**Published:** 2020-01-23

**Authors:** Katarina Baralić, Aleksandra Buha Djordjevic, Katarina Živančević, Evica Antonijević, Milena Anđelković, Dragana Javorac, Marijana Ćurčić, Zorica Bulat, Biljana Antonijević, Danijela Đukić-Ćosić

**Affiliations:** Department of Toxicology “Akademik Danilo Soldatović”, University of Belgrade—Faculty of Pharmacy, Vojvode Stepe 450, 11221 Belgrade, Serbia; aleksandra.buha@pharmacy.bg.ac.rs (A.B.D.); kajaziv93@gmail.com (K.Ž.); evica.antonijevic@pharmacy.bg.ac.rs (E.A.); millena.andjelkovic@gmail.com (M.A.); dragana.javorac@pharmacy.bg.ac.rs (D.J.); marijana.curcic@pharmacy.bg.ac.rs (M.Ć.); zorica.bulat@pharmacy.bg.ac.rs (Z.B.); biljana.antonijevic@pharmacy.bg.ac.rs (B.A.); danijela.djukic.cosic@pharmacy.bg.ac.rs (D.Đ.-Ć.)

**Keywords:** phthalates, bisphenol A, mixture toxicity, endocrine disruptors, subacute exposure

## Abstract

Phthalates and bisphenol A, classified as endocrine disruptors, have weak estrogenic, anti-androgenic properties, and affect thyroid hormone regulation. The aim of this study on male rats was to compare the subacute toxic effects of low doses of single compounds (bis (2 –ethylhexyl) phthalate (DEHP), dibutyl phthalate (DBP), and bisphenol A (BPA)) with the effects of their mixture through different biochemical, hormonal, and hematological parameters. Rats were divided into five experimental groups: Control (corn oil), DEHP (50 mg/kg b.w./day), DBP (50 mg/kg b.w./day), BPA (25 mg/kg b.w./day), and MIX (50 mg/kg b.w./day DEHP + 50 mg/kg b.w/day DBP + 25 mg/kg b.w./day BPA). Animals were sacrificed after 28 days of oral treatment and blood was collected for further analysis. The results demonstrated that the mixture produced significant changes in lipid profile, liver-related biochemical parameters, and glucose level. Furthermore, the opposite effects of single substances on the thyroxine level have been shown in comparison with the mixture, as well as a more pronounced effect of the mixture on testosterone level. This study contributes to the body of knowledge on the toxicology of mixtures and gives one more evidence of the paramount importance of mixture toxicity studies, especially in assessing the endocrine disruptive effects of chemicals.

**Highlights**

- More adverse effects induced by the mixture compared to the single substances
- Significant changes in lipid profile, liver-related biochemical parameters, and glucose level
- Opposite effects of single substances on the thyroxine level in comparison with the mixture
- More pronounced effect of the mixture on testosterone level
- Mixture toxicity studies are of primary importance for endocrine disruptors

## 1. Introduction

In real-life scenarios, the general population experiences uncontrolled multi-chemicals low-dose exposure from many different sources [1,2,3,4,5]. Epidemiological and biomonitoring studies have shown that exposure to chemical mixtures is associated with various adverse health effects as a consequence of different types of toxicity, such as neurotoxicity, cardiotoxicity, hematotoxicity, nephrotoxicity, hepatotoxicity, as well as endocrine disruption [2,5,6,7,8,9,10]. Exogenous compounds with the potential to disturb hormonal regulation and normal endocrine system, consequently affecting health and reproduction in animals and humans, are often referred to as endocrine disruptors (ECDs) [11]. The prerogative for identification of the substance as EDC is the existence of reasonable evidence of a biologically plausible causal relationship between the endocrine activity and the induced adverse effect(s) seen in an intact organism, or a (sub)population [11]. This definition of EDCs was later endorsed by the Scientific Committee of the European Food Safety Authority [12]. Recent Expert Consensus Statement developed ten key characteristics that can be used to identify, organize, and utilize mechanistic data during EDCs evaluation [13]. However, there are some areas in which there is still a lack of scientific consensus, such as the existence of thresholds for EDCs, non-monotonic dose responses (NMDR), and critical windows of exposure, as recently stated in the report of Endocrine Disrupters Expert Advisory Group [14]. Another unresolved issue is the toxicity of EDCs mixtures. There is evidence that EDCs with similar modes of action (MoAs) can act together in an additive manner to produce more pronounced effects [15]. Additionally, there are studies on different experimental models that indicate the possibility of interaction between these chemicals, i.e., suggesting even synergism in their endocrine actions [7,16,17,18,19]. Therefore, the utilization of real-life risk simulation (RLRS) concept in determining the combined effects of such chemicals is essential [19]. 

Some of the already identified and well-established chemicals with endocrine-disrupting properties include organochlorine pesticides, polybrominated diphenyl ethers, polychlorinated biphenyls, toxic metals, as well as phthalates and bisphenol A [1,20,21,22,23]. The results from a recent nation-wide survey of the levels of environmental contaminants among pregnant women in France showed that these chemicals are quantifiable in virtually all individuals [24]. 

The current study will concentrate on the investigations of the combined effects of selected phthalates and bisphenol A. Phthalates have been used as softeners in PVC plastic and vehicles for fragrance in cosmetics [25], while bisphenol A has been used as a polymer in polycarbonate plastics, in food packaging, dental sealants, thermal receipts, etc. [26]. Because of their omnipresence, human exposure to these substances is ubiquitous and almost inevitable, mainly through the ingestion of chemical-contaminated food. These compounds are not covalently bound to plastic, often leach and contaminate foods processed or stored in plastic products [27]. Among the phthalates, bis(2-ethylhexyl) phthalate (DEHP), and dibutyl phthalate (DBP) have a common mode of action, but different active metabolites (monobutyl phthalate (MBP) versus mono-(2-ethylhexyl) phthalate (MEHP)) and are considered to have the greatest impact on development of metabolic disorders [28,29,30]. 

Studies on experimental animals have shown that phthalates and bisphenol A interfere with the endocrine system inducing developmental and reproductive toxicity [31,32]. These substances are considered to have weak estrogenic and anti-androgenic properties, mostly by competing with endogenous steroid hormones binding to receptors [33,34]. Moreover, recent interests are being directed to the effects of these substances on the thyroid tissue and thyroid hormone regulation, linking the changes in thyroid function with the consequential adverse reproductive health [35,36]. To investigate the effects of phthalates on the thyroid, in vivo rodent studies have been conducted to reveal the decrease in thyroid hormones after oral exposure to DEHP [35,37,38], DBP [39], and BPA [40,41]. This is in accordance with the human biomonitoring study from the national health and nutrition examination survey (NHANES, 2007–2008), which included 1346 adults (ages ≥20 years) and 329 adolescents (ages 12–19 years). In this study, measured urinary DEHP, DBP, and BPA metabolites could be linked with the decrease in thyroid hormones’ concentration (triiodothyronine (T3) and thyroxine (T4)). Conversely, in the same study, among the adolescents, DEHP metabolites could be connected with the increase in T3 concentration [42].

Having in mind that phthalates and bisphenol A coexist in natural environments, their combined effects require further investigation [31]. Reproductive toxicity of phthalates and bisphenol A has been investigated in binary and multicomponent mixtures, usually targeting male reproductive tract disorders, mostly after the perinatal exposure [31,43,44,45]. For example, it has been demonstrated that prenatal exposure to the mixture of DBP and DEHP alters fetal testosterone production and insl3 gene expression in a manner that resulted in cumulative dose-additive increases in reproductive tract malformations [44,46]. Furthermore, Howdeshell et al. (2008) reported a mixture experiment, including five phthalates, in which co-exposure of pregnant rats resulted in suppression of fetal testosterone production in a dose additive manner [44]. Similar dose additive effects have been reported for mixtures of phthalates with antiandrogenic pesticides of different mechanisms of action [44,47].

However, studies on the subacute toxicity of the mixture of DEHP, DBP, and BPA are, to the best of our knowledge, limited, if nonexistent, especially those considering the thyroid-disrupting effects. Therefore, the aim of this experimental study was to compare the effects of single compounds (DEHP, DBP, and BPA) with the effects of their mixture on different hematological, biochemical, and parameters of endocrine function in rats orally exposed to these chemicals for 28 days. 

## 2. Materials and Methods

### 2.1. Chemicals

Bis(2-ethylhexyl) phthalate (≥99.5% DEHP, Sigma-Aldrich-Chemie, Steinheim, Germany), dibutyl phthalate (98% DBP, Sigma-Aldrich-Chemie, Steinheim) and bisphenol A (97% BPA, Sigma-Aldrich-Chemie, Steinheim) were used in this study. All reagents and chemicals were of analytical grade quality or higher purity. Phthalates and bisphenol A were dissolved in corn oil. 

### 2.2. Animals

Male Wistar rats (*Rattus norvegicus*), weighing 150–200 g, were used for the experiment. Animals were housed under the standard controlled conditions (temperature (20–24 °C), light (12 h night: 12 h day), relative humidity of 35% to 60%. They were allowed free access to standard rat chow (purchased from The Veterinary Institute Subotica (Subotica, Serbia)) and tap water during the experiment. Animals were given seven days to acclimate to their new environment and experimental conditions. All experimental procedures were approved by the Ethical Committee on Animal Experimentation of the University of Belgrade, Faculty of Pharmacy (Ethical approval number: 323-07-11822/2018-05).

### 2.3. Study Design and Experimental Procedure

In this study, we have chosen to use only male rats having in mind all the evidence that both phthalates and bisphenol A are toxic to the male reproductive system [48,49]. Rats were randomly divided into five groups (*n* = 6): Control and four treated groups, of six animals each: (i) Control group (corn oil), (ii) DEHP (50 mg/kg b.w./day), (iii) DBP (50 mg/kg b.w./day), (iv) BPA (25 mg/kg b.w./day), (v) MIX (50 mg/kg b.w./day DEHP + 50 mg/kg b.w/day DBP + 25 mg/kg b.w./day BPA). Not more than six rats were used per group, in accordance with the 3Rs principle and good animal welfare. Body weights, as well as food and water consumption, were recorded daily and the dose administered each day was adjusted for the body weight. Treatment of all animals was performed by oral gavage 28 days. The doses selection was made according to the reported overall no-observed-adverse-effect level (NOAEL) for DBP (50 mg/kg b.w./day) [50], and NOAEL for the effects on the bodyweight for BPA (25 mg/kg b.w./day) [51]. The dose level for DEHP (50 mg/kg b.w./day) was chosen based on the studies regarding the effects on lipid metabolism [52] and glucose homeostasis [53]. Furthermore, although this dose is 10 higher from oral NOAEL of 5 mg/kg b.w./day [54] for development toxicity, it is 15 times lower from the dose that could cause adverse effects without inducing systematic toxicity (LOAEL) for androgen inhibition in pubertal rats (750 mg/kg) [55]. Oral administration of investigated substances was chosen to reflect human exposure to DEHP, DBP, and BPA, since it mostly occurs through food [29,46].

### 2.4. Blood Collection, Body and Organ Weights

On the 28th day rats were weighted and euthanized under light anesthesia, intraperitoneal ketamine (75 mg/kg b.w.)/xylazine (10 mg/kg b.w.) injection. Blood samples were collected by cardiac puncture after anesthetic administration. For biochemical and hormonal parameters, blood was collected in a centrifuge tube for serum separation. Serum was separated by centrifugation at 3000× *g* for 30 min and frozen (−20 °C) for biochemical assays and serum hormone level analysis. One aliquot of blood, collected in heparin vacutainers, was used for the determination of hematological parameters. At the end of the experiment, final body weights and weight gain for all animals were calculated and recorded. Bodyweight gain was calculated by the following equation:

BWG = (mf − mi)/mi(1)

In this equation, mf signifies final body weight, while mi signifies initial body weight. The organs were collected and weighed, and the relative organ weight was calculated by dividing organ weight with the final body weight.

### 2.5. Haematology Analysis

Hematological parameters were measured according to good laboratory practices by the MYTHIC 22 analyzer (Orphee Medical, Geneva, Switzerland). Multi-angle polarized scatters separation was used for white blood cell and dual-angle optical analysis for platelets count. The following hematological parameters were examined: White blood cell count (WBC) with WBC differential count (neutrophils (NEU), eosinophils (EOS), lymphocytes (LYM), basophils (BAS), and monocytes (MON)), red blood cell count (RBC), hemoglobin concentration (HGB), hematocrit (HCT), mean corpuscular volume (MCV), mean corpuscular hemoglobin (MCH), mean corpuscular hemoglobin concentration (MCHC), and platelet count (PLT).

### 2.6. Biochemical Analysis

All biochemical assays were performed with commercial reagents and according to the good laboratory practices on the Cobas C311 analyzer (Diagnostics Roche, Basel, Switzerland). The measured biochemical parameters in rat serum included triglycerides (TG), cholesterol, high-density lipoprotein (HDL), low-density lipoprotein (LDL), creatinine (CRE), uric acid (UA), total serum proteins (TP), albumin (ALB), direct bilirubin (DB), total bilirubin (TB), aspartate aminotransferase (AST), alanine aminotransferase (ALT), alkaline phosphatase (ALP), serum iron, calcium (Ca^2+^), inorganic phosphorus (PO_4_^3−^), magnesium (Mg^2+^), and chloride (Cl^−^). In order to identify the cause of liver damage the AST/ALT (the De Ritis ratio) ratio was calculated. 

### 2.7. Hormone Analysis

Serum samples were analyzed by electro-chemiluminescent immunoassay (ECLIA) tests by Cobas E411 analyzer (Diagnostics Roche, Basel, Switzerland). The measured hormonal parameters in rat serum included triiodothyronine (T3), thyroxine (T4), and testosterone. All assays were performed with commercial reagents and according to the good laboratory practices. 

### 2.8. Statistical Analysis

The statistical analyses were performed using GraphPad Prism 6 software (GraphPad Software, Inc., San Diego, California, USA). The normality of distribution for all parameters was tested using the Shapiro–Wilk method in all experimental groups. In the case of the normal distribution, a one-way analysis of variance (one-way ANOVA) was used to analyze the differences between the experimental groups. Fisher’s least significant difference (LSD) as a post-hoc test was performed. If the distribution was not normal, the non-parametric Kruskal–Wallis test was used for group differences along with the Dunn’s post-hoc test. A value of p < 0.05 was considered statistically significant.

## 3. Results

### 3.1. Body Weight Gain, Food and Water Consumption

Bodyweight gain (BWG) and percentage difference of BWG in four selected time points during the experiment are presented in Table 1. Body weight gain was significantly lower in all the treated groups compared to the control in all presented time points, with the exception of the DEHP group. In this group, bodyweight gain was significantly lower only after the first week of treatment. However, according to WHO Guidance document (Pesticide Residues in Food) of the WHO Core Assessment Group on Pesticide Residues [56], an adverse change in body weight or body weight gain is considered any weight above or below 10% of the control value. According to these criteria, as most of the observed changes in the BWG are out of the range ± 10%, they should be considered as adverse.

On the other hand, there was no significant difference in body weight gain between the MIX group compared to all the groups in which rats were receiving single substances for all presented time points.

### 3.2. Food and Water Consumption

**Figure 1:**
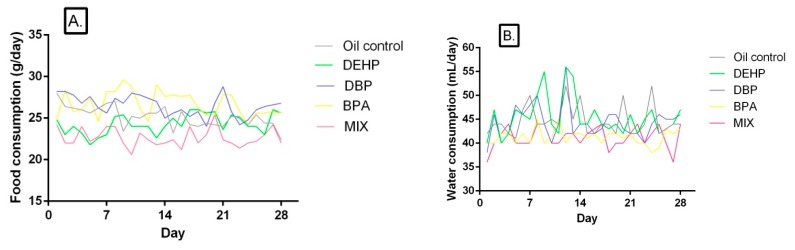
(**A**) Food (g/day) and (**B**) water (mL/day) consumption of rats exposed to DEHP, DBP, BPA, and their mixture each day for 28 days.

Food and water consumption of rats in the control and treated groups are given in Table 2 and Table 3, while daily food and water intake are presented in Figure 1B,C, respectively. The food consumption was significantly lower compared to the control in the MIX group in all four presented time points, while in the DEHP group it was lower only after the first week of treatment. The increase in food consumption was noted in the DBP and BPA groups after the third week of treatment. However, overall food consumption was statistically different in all treated groups compared to the control, lower in the DEHP and MIX groups, and higher in the DBP and BPA groups (Figure 1A). Furthermore, food consumption was significantly lower in the MIX group compared to both the DBP and BPA groups in all presented periods of time, while it was significantly different from the DEHP group only after the second and fourth presented period of treatment. Nevertheless, overall food consumption was significantly lower in the MIX group compared to all the other groups in which rats were being treated with single compounds. 

A significant decrease in overall water consumption was noted in BPA and MIX group compared to the control, as well as in the first three time points (Table 3). Water consumption was significantly decreased in the DBP group only in the third presented time point, and in the DEHP group after the fourth week of treatment. Overall water consumption was affected neither in the DEHP, nor in the DBP group (Figure 1B).

On the other hand, water consumption was significantly higher in the DEHP and DBP groups compared to the MIX in all four presented time points, except for the third week, after which there was no significant difference for the DBP group compared to the MIX. After both the first and the second week, water consumption was significantly higher in the BPA group compared to the MIX, but lower compared to the oil control. However, there was no significant difference between the BPA and MIX group after the third and fourth week. As for the overall water consumption, it was significantly higher in the DEHP and DBP groups compared with the MIX, while there was no significant difference between the BPA and MIX group. 

### 3.3. Relative Organ weight

Relative organ weights are presented in Table 4. There was a significant increase in liver relative weight in rats receiving DEHP (24%), as well as in the MIX group (17%) in comparison with the control. Additionally, liver relative weight was significantly lower in both the DBP and BPA groups compared to the MIX, while there was no significant difference between DEHP and MIX. Spleen relative weight was significantly lower in the MIX group compared to the control (−23%), while only in the DBP group it was significantly lower when compared to the MIX, which was also the case with brain relative weight, significantly lower only in the DBP group compared to the MIX. Kidney relative weight was higher in the MIX group compared to the control, as well as compared to the DBP and BPA groups. Thymus relative weight was significantly higher in the DEHP group compared to the MIX. Although not statistically different in all the single substances groups, a slight increase in lung, kidneys, testicles, brain and thyroid gland relative weight was noted in the MIX group compared to all the single substances, while a decrease was noted in spleen relative weight in comparison with all the single substances.

### 3.4. Haemotological Parameters

Hematological parameters measured in the present study are presented in Table 5. In rats receiving DEHP, a significant increase in WBC, LYM, and MON was noted compared to the control. The DBP group has shown a significant increase in LYM and WBC, while in the BPA group, RBC, HGB, HCT, and MCHC were elevated compared to the control. The changes in most of the parameters were noted in the MIX group, in which WBC, LYM, NEU, MON, RBC, HGB, HCT, and MCHC were elevated compared to the control. When compared to the groups that were receiving single substances, a significant decrement was noted in the BPA group compared to the MIX in WBC, LYM, and MON, while in the DBP group RBC and HGB were lower compared to the MIX. Hematocrit and MCHC were significantly lower in the DEHP and DBP groups compared to the MIX. There were no significant differences in EOS, BAS, MCH, and PLT in rats of all the four groups. Having in mind that only the values of MON and NEU in MIX group are different from the individual values, this could be attributed to the additive effect of the investigated substances, while, in the case of other parameters, values of the MIX are equal or similar to the values of some of the individual component groups.

### 3.5. Serum Biochemistry Parameters

Serum biochemistry parameters after 28 days of exposure to the investigated substances and their mixture are presented in Table 6. More parameters were significantly different in the MIX group in comparison with control than in the groups receiving single substances. 

**Figure 2:**
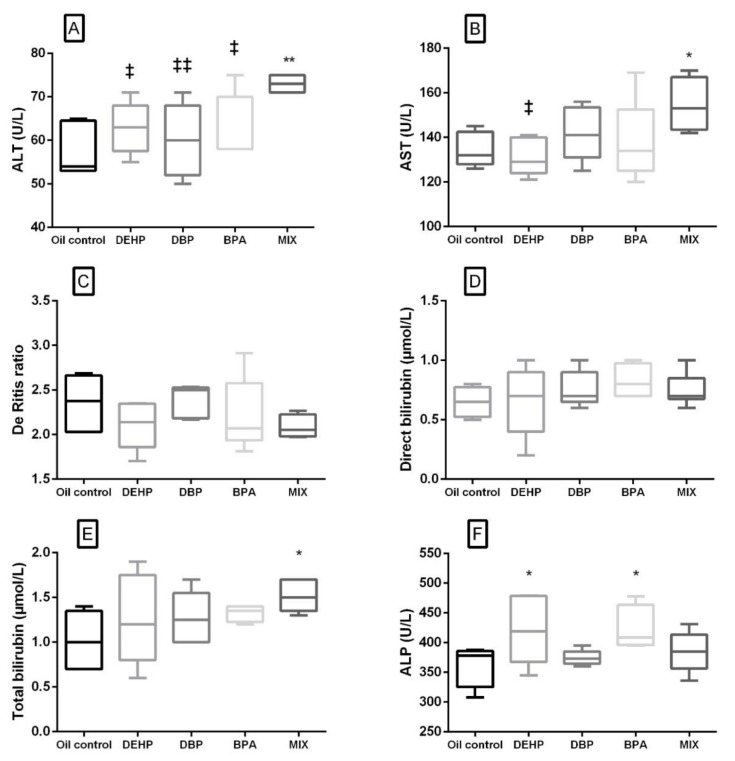
Liver-related serum biochemical parameters after 28 days of oral exposure to single investigated substances and their mixture (MIX). Each box plot represents interquartile range (25–75th percentile), the line within the box represents median value, and ends of the whiskers represent the minimum and maximum values within the group. (**A**) ALT activity (U/L), (**B**) AST activity (U/L), (**C**) the De Ritis ratio (AST/ALT), (**D**) direct bilirubin concentration (μmol/L), (**E**) total bilirubin concentration (μmol/L), (**F**) ALP activity (U/L) ((DEHP, DBP and BPA) * *p* < 0.05, ** *p* < 0.01 (compared to the control), ^‡^ *p* < 0.05, ^‡‡^ *p* < 0.01 (compared to the MIX group).

Liver-related serum biochemistry parameters are presented in Figure 2. In the MIX group, significant elevation of ALT, AST and total bilirubin was noted compared to the control (Figure 2A,B,E). Out of the liver-related parameters, in the DEHP and BPA groups, a significant increase was noted only in ALP activity (Figure 2F). When compared to the MIX group, ALT activity was significantly lower in the groups that were receiving single substances, while AST was significantly lower in the DEHP group only (Figure 2A,B). There was no significant difference in The De Ritis ratio and direct bilirubin level in none of the experimental groups in comparison with the control, as well as between the single substances and the MIX group (Figure 2C,D). 

In the MIX group, there was a significant elevation of the urea level compared to the control. Having in mind that there were no significant changes in this parameter in single substance groups, this could suggest the additive effect of the investigated substances. However, there was no significant difference between the urea level in the DEHP, DBP, and BPA groups compared to the MIX.

**Figure 3:**
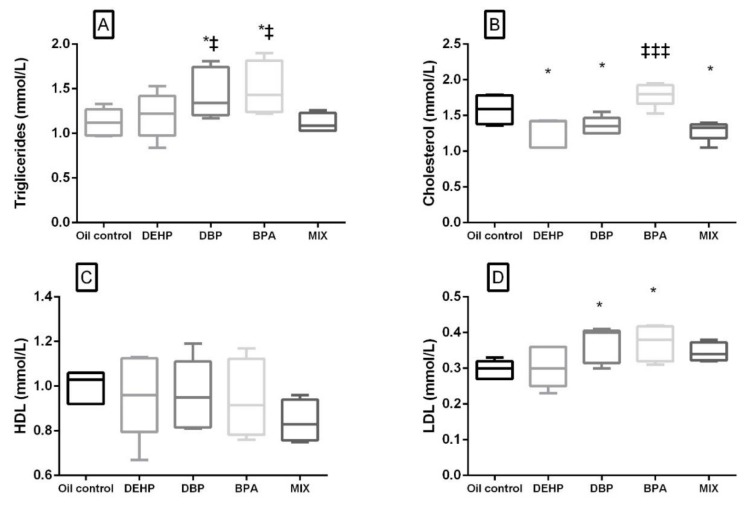
Lipid profile after 28 days of oral exposure to single investigated substances (DEHP, DBP, and BPA) and their mixture (MIX). Each box plot represents interquartile range (25–75th percentile), the line within the box represents median value, and ends of the whiskers represent the minimum and maximum values within the group. (**A**) Triglycerides (mmol/L), (**B**) cholesterol (mmol/L), (**C**) HDL (mmol/L), (**D**) LDL (mmol/L). * *p* < 0.05 (compared to the control), ^‡^ *p* < 0.05, ^‡‡‡^ *p* < 0.001. (compared to the MIX group).

A significant increase in the glucose level compared to the control was noted not only in the MIX, but in the DEHP and DBP groups as well. There was no significant difference in the glucose level in the BPA group compared to the control, but it was significantly lower when compared with the MIX group. Lipid profile parameters are presented in Figure 3. A significant decrease in the cholesterol level was noted in the DEHP, DBP, and MIX groups (Figure 3B), while LDL and TG were significantly elevated in DBP and BPA groups comparison to the control (Figure 3A,D). There was no significant difference in HDL levels in all the groups compared to the control (Figure 3C). When compared to the MIX, cholesterol was significantly higher in the BPA group, while TG was significantly increased, not only in the BPA, but also in the DBP group. 

### 3.6. Hormone Parameters

Hormone parameters measured in this study are presented in Table 7. In all experimental groups, there was no significant difference in the T3 level compared to the control. However, a significant decrease in the T4 serum level was detected in DBP and BPA group, −30% and −24% compared to the control, respectively. The opposite observation was made in the MIX group, where the T4 serum level was increased (28.5% in comparison with the control). A significant decrease in the T3/T4 ratio was noticed only in the MIX group (−32% in comparison with the control). When compared to the MIX group, in all three groups receiving single substances T4 was significantly lower, and, consequently, the T3/T4 ratio was significantly increased in these groups. There was no significant difference in the T3 level in none of the groups receiving single substances in comparison with the MIX group. 

In the groups receiving single substances, the testosterone level was lower, but not significantly. Nevertheless, in the MIX group, a notable decrease in this parameter was observed compared to the control. However, there was no significant difference in testosterone levels between the groups receiving single substances in comparison with the MIX group. 

## 4. Discussion

In this experimental study on rats, subacute toxic effects of single compounds (DEHP, DBP, and BPA) and their mixture were investigated through different biochemical, hormonal, and hematological parameters.

### 4.1. Body Weight Gain, Food and Water Consumption

Although the given dose of BPA is considered the NOAEL for the effects on body weight, in this study, a decrease in body weight was shown in almost all presented time points, as well as for the overall body weight gain. In their study, Miao et al. (2008) stated that the ability of BPA to put the stress on rats resulting in a decrease in their weight by depressing the appetite and, thus, reducing the food intake [57]. However, in our study, despite the overall food consumption in the DBP and BPA groups being significantly higher compared to the control, lower body weight gain was recorded in these groups compared to the control in all presented time periods. Phthalates and bisphenol A are known to be obesogens. During critical windows of development, *in utero* exposure to BPA, DEHP, and DBP can lead to obesity later in life, through various mechanisms, such as disturbances of the methylation process and changes in the histone structure which consequently affect gene expression in the case of phthalates [58] and alteration of energy and amino-acid metabolism in the case of BPA [59]. However, it should be emphasized that, in our experiment, exposure was non-developmental. Thus, it could be speculated that some other mechanisms were behind observed increased anabolic reactions. The decrease in water consumption as well as in food consumption was observed in the MIX group and was followed by the significantly lower body weight gain when compared to the controls. However, there was no significant difference in body weight gain between the MIX group and DEHP, DBP, and BPA groups, since body weight gain was similarly lower in all the treated groups compared to the control. Furthermore, a decrease in water consumption, i.e., possible dehydration can be one of the explanations for the observed elevation in erythrocyte count, HGB, HCT, MCHC, as well as serum urea level. We can assume that the type of dehydration was isotonic, in which both water and electrolytes are lost proportionally, such that the serum electrolytes concentration maintains normal serum osmolality.

### 4.2. Inflammation

It was noted that DEHP, DBP, and MIX groups caused a significant increase in the level of lymphocytes, while there was no significant difference in the BPA group compared to the control. This, along with the CRP level, significantly elevated in these groups as well, could be linked to the low-grade systemic inflammation caused by the investigated substances. This is in accordance with the results obtained in the study, in which rats were orally exposed to 0, 5, 50, and 500 mg/kg b.w./day DEHP for eight weeks. This study has demonstrated the increased inflammatory factors (IL-1β and TNF-α) and concluded that the inflammation may play a regulatory role in lipid metabolic disorders induced by DEHP [52]. Furthermore, a study on rats reported a high correlation between the concentration of anti-thyroid peroxidase antibodies and the degree of lymphocytic infiltration of the thyroid gland after the five weeks of DBP exposure at 5 and 50 mg/kg b.w./day doses [39,60].

### 4.3. Thyroid Hormones

In our study, thyroid function was investigated by measuring T3 and T4 serum content. A significant negative association was found in the DBP group for the T4 level, as well as in the BPA group. In a study on rats by Wu et al. (2017) the decrease in the T3 and T4 levels was demonstrated after five weeks of oral exposure to the same dose of DBP (50 mg/kg/day) [39]. Conversely to our results, the increase in the T4 level was noted in a study in which rats were orally treated with a slightly higher dose of BPA than in our experiment (40 mg/kg b.w.) for 15 days [61]. In the same study, the T3 level remained unchanged, while the T3/T4 ratio was decreased in a treated group. As a conclusion, the authors suggested that the peripheral metabolism of thyroid hormone was affected by BPA exposure [61]. It can be suggested, based on the results of our study that after four weeks of exposure to BPA, which corresponds to three years of exposure in humans according to Sengupta (2013) [62], the defense mechanisms are no longer capable of preventing the BPA-induced thyroid disruption. Interestingly, however, our results did not show any significant change in the T4 level in the DEHP group. This can possibly be a result of the defense mechanisms activation under the influence of lower DEHP doses, which are surpassed in the presence of the higher doses and longer DEHP exposure. Similarly to our study, Sun et al. (2018) observed no difference in the T4 and T3 level at the same dose as in our experiment (50 mg/kg b.w.) but decreased the T3 and T4 serum levels after the exposure to much higher dose (500 mg/kg b.w.) of DEHP for 28 days [38]. Another study that demonstrated the decrease in thyroid hormones at higher DEHP doses (250, 500, and 750 mg/kg b.w.) indicates that DEHP could reduce thyroid hormones through influencing biosynthesis, biotransformation, biotransport, receptor levels, as well as the metabolism of thyroid hormones [63]. On the other hand, our results have demonstrated significantly increased levels of T4 in the MIX group when compared to the control with mixture showing unexpected opposite pattern to the effect of single substances. Similar results were observed in a study in which rats were given the same DBP dose in combination with thyroglobulin immunization [39]. In this study, lower T3 and T4 levels were demonstrated in groups receiving only DBP, while a mixture of DBP and thyroglobulin expressed an opposite effect, a significant increase in these hormones. Authors have suggested a possibility of different mechanisms influencing the production of thyroid hormones depending on whether DBP is present alone or in combination with thyroglobulin, demonstrating that oxidative stress induced by thyroglobulin plays an important role in the DBP-exacerbated effect. [39]. Our study has shown a similar pattern in the MIX group, in which the T4 level was increased, along with the decrease in the T3/T4 ratio. Hence, the ability of BPA to induce oxidative stress [64] might be one of the explanations for the observed effects of mixtures. Furthermore, it could be speculated that the increase in T4 in the MIX group could be attributed to the well-known NMDR for EDCs [14,19,65], especially in the case of BPA [23,66]. Therefore, it is reasonable to presume the possibility of the shift in the dose-response axis direction due to the dose addition of components of the mixture. However, further studies are needed to investigate the mechanisms behind the combined effects of these substances on thyroid function, with an assessment of various cytokines and chemotactic molecules in thyroid tissue, as well as oxidative stress markers.

### 4.4. Lipid Profile

In our study, a decrement in the cholesterol level was observed in the DEHP, DBP, and MIX groups compared to the control, while the cholesterol level in BPA was significantly higher in comparison with the MIX group. Triglyceride concentration was significantly elevated in the DBP and BPA groups compared to the control. The hypo-cholesterol level may be explained by the possible impairment of the liver function, in the case of the DEHP group, in which the increase in the relative liver weight was observed, along with the increase in ALP activity compared to the control. The decrement in the cholesterol level might also be connected with the decrease in the T4 level and altered thyroid function present in the DBP group [67]. Furthermore, changes in cholesterol and the TG level might be connected with the inflammation noticed in the DBP and MIX groups, generally accepted as one of the key links in the process of lipid metabolic disorders [52]. Our findings can be compared to the results of a 13-week study on rats, in which Majeed et al. (2017) demonstrated a decrease in serum cholesterol levels upon the administration of the same dose of DBP (50 mg/kg) [68]. In the BPA group, a slight, but not significant elevation of cholesterol was observed, while the LDL and TG level were significantly elevated. Abdel-Wahab et al. (2014) also reported the increase in LDL and TG upon the oral administration of lower BPA dose (10 mg/kg/day) for the same period of time. In this study, a significant increase in total cholesterol was observed [69]. The function of thyroid hormones is energy metabolism regulation and, having in mind that the T4 level was significantly decreased in DBP and BPA group, can be connected with the elevated triglyceride level in the aforementioned groups. No significant change was observed in TG, LDL, and HDL level in comparison with the control upon the administration of DEHP for 28 days. These results are consistent with the results of the study in which peripubertal male rats were exposed to 7.5 and 75 mg/kg b.w./day for 30 days [70]. In this study, no significant change was noted in the same lipid profile parameters that were investigated in our study [70]. However, in their eight-week study on rats, Zhou et al. (2019) demonstrated the increase in this parameter, as well as LDL and HDL, at a higher DEHP dose (500 mg/kg) [52].

### 4.5. Liver-Related Biochemical Parameters

Agents that cause peroxisome proliferation in the liver might also cause thyroid hyperactivity, and it is commonly assumed that the two actions are mechanistically linked [37]. Kim at al. (2018) demonstrated that exposure to low-dose DEHP in juvenile rats from young ages to maturity, may induce proliferative changes in thyroid tissues without changes in liver histology [35]. However, in our study, statistically significant elevated values of total bilirubin, AST and ALT were noted in the MIX group, as well as a slight, but not statistically significant decrease in the De Ritis ratio. This, along with the increase in the relative liver weight, suggests the possible mixture-induced hepatocellular injury and intrahepatic biliary obstruction, which can be connected with PPAR and ER agonists [9]. In the DEHP group, a significant increase has been noted only in ALP activity, along with the increase in relative liver weight. Various studies have shown that PPARα agonists, including DEHP and DBP, suppressed hepatic apoptosis in low doses, while elevated the frequency of apoptotic hepatocytes in higher doses [71,72,73,74]. Another parameter connected with the liver function in the present study was albumin. However, there was no significant change observed in its concentration compared to the control in any of the groups. Nevertheless, having in mind that dehydration (possibly present in the DBP and MIX groups) could lead to the higher concentrations of this parameter in serum, there is a chance that albumin could be slightly lower in these groups if the blood volume were not affected.

### 4.6. Glucose Level

The present study has demonstrated a significant increase in the glucose level compared to the control in DEHP, DBP, and MIX groups, while the glucose level was significantly lower in the BPA group compared to the MIX. In support of our results, Srinivasan et al. (2011) showed the connection between DEHP 30-day rat oral exposure and the decrement of both testosterone and insulin levels at the dose 100 mg/kg, connecting the DEHP exposure to the higher glucose level in rat serum [75]. Impairment of insulin secretion can be associated with several metabolic changes in liver, leading to disruption of glucose homeostasis, having in mind this hormone stimulates glucose utilization and storage and represses its synthesis and release by this tissue [76]. High glucose levels can often be accompanied by lipid abnormalities such as increased triglycerides and LDL, which is, in the case of our study, present only in the DBP group. It can be assumed that the exposure was not long enough to induce consistent results regarding these changes in other groups. The effect of the 100 mg/kg 30 days DEHP rat oral exposure on the glucose level has been confirmed by Rajesh et al. (2013), connecting the DEHP-induced insulin deficiency and the defective insulin signal transduction at the molecular level. However, in this study, the lower dose (10 mg/kg) of DEHP has shown no effect on the glucose level [77]. In a 13-week study on rats, Majaeed et al. (2017) investigated the effect of 10 and 50 mg/kg b.w. DBP on the serum glucose level. In this study, an increase in serum glucose was observed in an inverted U-shaped non-monotonic fashion. Although the glucose level was high in both treated groups, it was more affected in the group with the lower dose [68]. The results of the study in which mice were exposed to lower BPA concentrations (0.5 and 2 mg/kg) for four weeks were contrary to our results, demonstrating that BPA dose-dependently increased blood glucose levels in the tested groups compared with the control [78]. It can be speculated that the NMDR can be the answer to these discrepancies as well, although further studies, including those dealing with more dose levels, are needed in order to elucidate the effects of BPA on glucose homeostasis.

### 4.7. Antiandrogen Activity and Serum Testosterone Level

In the present study, the serum testosterone level was measured as a marker of androgen activity. Our results have indicated a significant decrease in this parameter in comparison with the control only in the MIX group, while there was no significant difference upon the administration of single compounds (DEHP, DBP, BPA) for 28 days, although a slightly decreasing trend can be observed. The more pronounced effect of the mixture on the testosterone level can be explained by the possible toxicokinetic interactions between the substances present in the mixture. These interactions were confirmed in an acute toxicity study on mice, which explored the influence of a single dose of DEHP injected subcutaneously (3, 9, and 18 mg) on the deposition of BPA received as a food supplement (50 μg/kg 14C–BPA). The results of this study indicated that DEHP increases the deposition of BPA in the epididymis of male mice compared to the control group [79]. The effects of the single phthalate on the testosterone production have been tested in various studies, but mostly after the prenatal exposure. Furr et al. (2014) investigated the effect of various phthalates, including DEHP and DBP, on the postnatal fetal testosterone production, demonstrating the decrease in the testosterone level after the administration of both DBP and DEHP in the dose of 100 mg/kg to pregnant dams from gestational day (GD) 14 to 18 [80]. Bisphenol A effect on the testosterone level has been tested on prepubertal rats after subcutaneously administered BPA (0, 20, 100, and 200 mg/kg/day) for six weeks. This study has demonstrated the decrease in the testosterone level only after higher doses (100 and 200 mg/kg/day), in accordance with the results in our study [81]. Other studies have shown that BPA inhibits steroidogenesis in the rat testis and reduces testosterone secretion, thus inhibiting the activity of GnRH neurons, and lowering the expression of steroidogenic enzymes [13,82]. Jin et al. (2013) concluded that BPA orally administrated to the rats resulted in an impairment of spermatogenesis caused by stopping germ cells meiosis process and thus activating the apoptosis pathway in germ cells [83]. Dose additive effects have been shown for the phthalate mixture based on their effect on fetal testosterone production. In their experimental study on rats, Howdeshell et al. (2015) indicated the dose additive effects of the developmental exposure to a 5-phthalate mixture (benzyl butyl phthalate (BBP), di(n)butyl (DBP), bis (2 –ethylhexyl) phthalate (DEHP), di-isobutyl phthalate (DiBP) and dipentyl (DPP) phthalate). Authors suggested that the suppression of the steroidogenic pathway and insl3 gene expression in rat fetuses are given as the main reason for the impairment of the postnatal reproductive development in male rats [84]. Other authors listed decreased supply of cholesterol as one of the reasons phthalates affect steroidogenesis [85], which could be compared to our results having in mind that the cholesterol level was significantly lower in the MIX group as well as testosterone serum level. Androgenic activity was the main focus of a few studies which investigated the toxic effects of different plasticizer mixtures. Zhang et al. (2013) demonstrated that, under combined DBP and BPA treatment, the expression levels of sex hormone genes were higher compared to the control group, suggesting an additive or a synergistic effect [31]. The additive effect was also confirmed in mixture experiments with three phthalates (BBP, DBP, and DEHP) in combination with other antiandrogens (vinclozolin, procymidone, linuron, and prochloraz) [47]. Hence, it can be speculated that substances in the present mixture produced additive effects on the levels of testosterone, or even exerted toxicodynamic interactions. However, one of the main limitations of our study design is certainly the inability to assess both toxicokinetic and toxicodynamic interactions between the chemicals in the mixture. This being said, further studies assessing the toxicity of multiple doses of substances and mixtures are essential to confirm or infirm the suggested mixture effects.

## 5. Conclusions

In conclusion, this study demonstrated that a mixture of low doses of DEHP, DBP, and BPA produced significant changes in body weight gain, food, and water consumption, thyroid hormone and testosterone levels, lipid profile, liver-related biochemical parameters, and the glucose level. Some of these effects, especially those connected to endocrine disruption, were not observed in groups treated with single compounds. Overall, it could be concluded that the more pronounced effects observed at certain parameters with mixture exposure are due to the increased total exposure amount, suggesting the dose addition. The results of the study challenge the results of toxicity studies of single chemicals and further contribute to the understanding of the health effects posed by real-life exposure to chemical mixtures.

## Figures and Tables

**Table 1:** Bodyweight gain in rats orally exposed to DEHP, DBP, BPA, and their mixture (MIX) for 28 days

Group	Parameter	1st Week	2nd Week	3rd Week	4th Week
**Oil Control**	BWG	0.4344 ± 0.05996	0.8265 ± 0.1092	1.151 ± 0.1708	1.425 ± 0.2461
DEHP	BWG	0.3496 ± 0.02875 *	0.7347 ± 0.09337	1.077 ± 0.1378	1.327 ± 0.2461
	% to control	−20%	−11%	−6%	−7%
DBP	BWG	0.2826 ± 0.05433 ***	0.5559 ± 0.05379 ***	0.8089 ± 0.06926 ***	1.016 ± 0.08851 ***
	% to control	−35%	−33%	−30%	−29%
BPA	BWG	0.3228 ± 0.03756 ***	0.7001 ± 0.09798 *	0.9503 ± 0.1422 *	1.192 ± 0.1682 *
	% to control	−26%	−15%	−17%	−16%
MIX	BWG	0.3190 ± 0.03681 ***	0.6459 ± 0.04416 **	0.9237 ± 0.3505 **	1.147 ± 0.1048 **
	% to control	−26%	−22%	−20%	−19%

* *p* < 0.05, ** *p* < 0.01; *** *p* < 0.001. One-way ANOVA with LSD post-hoc test/Kruskal–Wallis test with Dunn’s post-hoc test. Values are presented as the means ± SD (*n* = 6). BWG: Body weight gain, DEHP: Bis (2-ethylhexyl) phthalate (50 mg/kg b.w.), DBP: Dibutyl phthalate (50 mg/kg b.w.), BPA: Bisphenol A (25 mg/kg b.w.), MIX: Mixture (50 mg/kg b.w. DEHP + 50 mg/kg b.w DBP + 25 mg/kg b.w BPA).

**Table 2:** Food consumption of rats orally exposed to DEHP, DBP, BPA, and their mixture (MIX) for 28 days.

Group	Parameter	1st Week	2nd Week	3rd Week	4th Week	Overall Food Consumption
Oil Control	Food consumption (g)	26.13 ± 1.451	25.9 ± 1.010	25.5 ± 0.5477	26.17 ± 1.169	25.16 ± 1.261
DEHP	Food consumption (g)	23.50 ±1.871 **	24.83 ± 2.483 ^‡^	24.27 ± 2.439	25.27 ± 0.7659 ^‡‡‡^	24.06 ± 1.796 ** ^‡‡‡^
	% to control	−10	−4	−5	−3	−4
DBP	Food consumption (g)	25.67 ± 1.211 ^‡‡^	25.67 ± 0.0165 ^‡‡^	27.47 ± 1.840 * ^‡‡‡^	27.13 ± 0.9933 ^‡‡‡^	26.52 ± 1.243 ** ^‡‡‡^
	% to control	−2	−1	8	4	5
BPA	Food consumption (g)	26.87 ± 1.633 ^‡‡‡^	27.10 ± 1.435 ^‡‡‡^	27.97 ± 1.417 * ^‡‡‡^	25.43 ± 1.023 ^‡‡‡^	26.70 ± 1.510 ** ^‡‡‡^
	% to control	3	5	10	−3	6
MIX	Food consumption (g)	23.17 ± 1.472 **	22.50 ± 1.871 **	22.57 ± 1.023 **	22.17 ± 1.169 **	22.66 ± 1.102 **
	% to control	−11	−13	−11	−15	−10

* *p* < 0.05, ** *p* < 0.01 (compared to the control), ^‡^ *p* < 0.05, ^‡‡^ *p* < 0.01, ^‡‡‡^ *p* < 0.001 (compared to the MIX group). One-way ANOVA with LSD post-hoc test. Values are presented as the means ± SD (n = 6). DEHP: Bis (2-ethylhexyl) phthalate (50 mg/kg b.w.), DBP: Dibutyl phthalate (50 mg/kg b.w.), BPA: Bisphenol A (25 mg/kg b.w.), MIX: Mixture (50 mg/kg b.w. DEHP + 50 mg/kg b.w DBP + 25 mg/kg b.w BPA).

**Table 3:** Water consumption of rats orally exposed to DEHP, DBP, BPA, and their mixture (MIX) for 28 days.

Group	Parameter	1st Week	2nd Week	3rd Week	4th Week	Overall Water Consumption
Oil Control	Water consumption (mL)	45.20 ± 0.8367	43.80 ± 0.8367	46.20 ± 1.924	44.20 ± 0.8367	44.68 ± 3.198
DEHP	Water consumption (mL)	44.60 ± 1.140 ^‡‡‡^	44.20 ± 0.8367 ^‡‡‡^	45.20 ± 1.304 ^‡‡‡^	46.80 ± 0.8367 ** ^‡‡‡^	45.43 ± 4.095 ^‡‡‡^
	% to control	−1	1	−2	6	2
DBP	Water consumption (mL)	44.20 ± 1.095 ^‡‡‡^	44.00± 0.7071 ^‡‡‡^	41.40 ± 1.440 ***	45.20 ± 0.8367 ^‡‡^	44.36 ± 3.551 ^‡‡^
	% to control	−2	0	−10	2	−1
BPA	Water consumption (mL)	41.60 ± 1.817 *** ^‡‡^	40.08 ± 1.183 *** ^‡^	41.50 ± 0.8944 ***	43.00 ± 1.531	41.29 ± 1.607 **
	% to control	−8	−8	−10	−3	−8
MIX	Water consumption (mL)	39.20 ± 1.304 ***	39.40 ± 1.140 ***	41.40 ± 0.8944 ***	43.00 ± 1.551	41.18 ± 2.294 ***
	% to control	−13	−10	−10	−3	−8

** *p* < 0.01, *** *p* < 0.001 (compared to the control); ^‡^ *p* < 0.05, ^‡‡^ *p* < 0.01, ^‡‡‡^ *p* < 0.001 (compared to the MIX group). One-way ANOVA with LSD post-hoc test. Values are presented as the means ± SD (*n* = 6). DEHP: Bis (2-ethylhexyl) phthalate (50 mg/kg b.w.), DBP: Dibutyl phthalate (50 mg/kg b.w.), BPA: Bisphenol A (25 mg/kg b.w.), MIX: Mixture (50 mg/kg b.w. DEHP + 50 mg/kg b.w DBP + 25 mg/kg b.w BPA).

**Table 4:** Relative organ weights (ROW) of rats orally exposed to DEHP, DBP, BPA, and their mixture (MIX) for 28 days.

Group	Parameter	Liver	Lungs	Heart	Kidneys	Testes	Spleen	Thymus	Brain	Thyroid Gland
Oil Control	ROW	3.448 ± 0.3311	0.3917 ± 0.04355	0.2833 ± 0.03266	0.6067 ± 0.03615	0.8117 ± 0.1137	0.2000 ± 0.02098	0.1967 ± 0.03011	0.4950 ± 0.05822	0.02600 ± 0.003847
DEHP	ROW	4.266 ± 0.2100 ***	0.3560 ± 0.0251	0.3160 ± 0.02302 *	0.6500 ± 0.05701	0.7880 ± 0.1011	0.1800 ± 0.02915	0.2260 ± 0.04615^‡^	0.4940 ± 0.03578	0.02400 ± 0.002236
	% to control	24	−9	12	7	−3	−10	15	0	−8
DBP	ROW	3.560 ± 0.2754 ^‡‡^	0.3440 ± 0.05413	0.3160 ± 0.02302	0.600 ± 0.01414 ^‡‡^	0.7860 ± 0.08264	0.1920 ± 0.03564 ^‡^	0.1720 ± 0.01483	0.4580 ± 0.01643 ^‡^	0.02250 ± 0.001915
	% to control	3	−12	12	−1	−3	−4	−13	−7	−13
BPA	ROW	3.390 ± 0.1920 ^‡‡‡^	0.3480 ± 0.04147	0.2720 ± 0.02588	0.6040 ± 0.03782^‡^	0.8120 ± 0.07120	0.1860 ± 0.03362	0.1680 ± 0.02387	0.4660 ± 0.03050	0.02260 ± 0.001517 *
	% to control	−2	−11	−4	0	0	−7	−15	−6	−17
MIX	ROW	4.032 ± 0.2513 **	0.4167 ± 0.03327	0.3017 ± 0.01602	0.6783 ± 0.05981 *	0.8467 ± 0.08937	0.1550 ± 0.01517 **	0.1767 ± 0.03445	0.5183 ± 0.03656	0.02467 ± 0.001862
	% to control	17	6	6	12	4	−23	−10	5	−5

* *p* < 0.05, ** *p* < 0.01, *** *p* < 0.001 (compared to the control), ^‡^ *p* < 0.05, ^‡‡^ *p* < 0.01, ^‡‡‡^ *p* < 0.001 (compared to the MIX group). One-way ANOVA with LSD post-hoc test/Kruskal–Wallis test with Dunn’s post-hoc test values are presented as the means ± SD (*n* = 6). ROW: Relative organ weight, DEHP: Bis (2-ethylhexyl) phthalate (50 mg/kg b.w.), DBP: Dibutyl phthalate (50 mg/kg b.w.), BPA: Bisphenol A (25 mg/kg b.w.), MIX: Mixture (50 mg/kg b.w. DEHP + 50 mg/kg b.w DBP + 25 mg/kg b.w BPA).

**Table 5:** Hematological parameters for male rats after 28 days of oral exposure to single investigated substances (DEHP, DBP, and BPA) and their mixture (MIX).

Parameter	Value Presentation	Oil Control	DEHP	DBP	BPA	MIX
WBC (10^9^/L)	Average	5.400	9.000 **	9.320 **	5.340 ^‡^	8.700 **
	SD	1.740	0.9850	1.450	1.419	0.8958
	% to control		67	73	−1	61
NEU (%)	Average	6.680	5.280	6.060	6.975	5.833
	SD	1.983	1.145	1.553	0.1500	0.7581
	% to control		−21	−9	4	−13
LYM (%)	Average	86.40	93.20 **	92.76 *	88.86	92.67 *
	SD	6.597	1.259	1.440	4.143	1.060
	% to control		8	7	3	7
MON (%)	Average	0.4333	0.6800	0.4600	0.7600	0.9200 *
	SD	0.1633	0.3768	0.2302	0.1140	0.3114
	% to control		57	6	75	112
EOS (%)	Average	0.4167	0.3000	0.3400	0.3000	0.2333
	SD	0.1472	0.2000	0.1342	0.1225	0.1966
	% to control		−28	−18	−28	−44
BAS (%)	Average	0.6000	0.5400	0.3800	0.6200	0.5167
	SD	0.2000	0.2881	0.08367	0.1483	0.2639
	% to control		−10	−37	3	−14
NEU (10^9^/L)	Average	0.4583	0.4683	0.5020	0.3700	0.6260*
	SD	0.1157	0.1516	0.1596	0.1089	0.05737
	% to control		2	10	−19	37
LYM (10^9^/L)	Average	4.810	8.205**	7.634 **	4.958^‡^	7.440 **
	SD	0.3540	0.9052	0.7413	1.335	2.180
	% to control		71	59	3	55
MON (10^9^/L)	Average	0.03667	0.0780 *	0.0600	0.0320^‡^	0.06333
	SD	0.02503	0.02280	0.03312	0.01095	0.04274
	% to control		113	64	−13	73
EOS (10^9^/L)	Average	0.0200	0.0280	0.0180	0.0260	0.01667
	SD	0.007071	0.01924	0.008367	0.008944	0.008165
	% to control		40	−10	30	−17
BAS (10^9^/L)	Average	0.03333	0.0492	0.0204	0.0531	0.04317
	SD	0.01325	0.02531	0.00666	0.0137	0.02740
	% to control		48	−39	59	30
RBC (10^12^/L)	Average	7.100	7.582	7.320^‡^	7.850 **	7.940 **
	SD	0.5571	0.2384	0.1775	0.3424	0.6403
	% to control		7	3	11	12
HGB (g/L)	Average	138.0	147	140.4	150.4 **	151.7 **
	SD	2.550	2.702	2.702	2.333	2.0101
	% to control		7	2	2	10
HCT (%)	Average	0.4400	0.4448 ^‡‡^	0.4476 ^‡‡^	0.4816 **	0.4940 **
	SD	0.02052	0.01706	0.0065	0.0101	0.01937
	% to control		1	2	9	12
MCV (fL)	Average	61.37	61.56	61.16	59.92	62.23
	SD	1.857	2.137	1.286	2.204	1.773
	% to control		0	0	−2	1
MCH (pg)	Average	19.12	18.98	19.18	19.40	19.10
	SD	0.5020	0.7120	0.4324	0.4848	0.3735
	% to control		−1	0	1	0
MCHC (g/L)	Average	313.2	313.0^‡^	313.6^‡^	317.4 *	317.5*
	SD	3.114	2.550	4.159	3.194	4.215
	% to control		0	0	1	1
PLT (10^9^/L)	Average	548.0	627.6	568.0	515.3	589.4
	SD	22.64	46.16	60.55	44.92	55.89
	% to control		15	4	−6	8

* *p* < 0.05, ** *p* < 0.01 (compared to the control); ^‡^ *p* < 0.05, ^‡‡^ *p* < 0.01 (compared to the MIX group); One-way ANOVA with LSD post-hoc test/Kruskal–Wallis test with Dunn’s post-hoc test. DEHP: Bis (2-ethylhexyl) phthalate (50 mg/kg b.w.); DBP: Dibutyl phthalate (50 mg/kg b.w.); BPA: Bisphenol A (25 mg/kg b.w.); MIX: Mixture (50 mg/kg b.w. DEHP + 50 mg/kg b.w DBP + 25 mg/kg b.w BPA); WBC: White blood cells, NEU: Neutrophils, LYM: Lymphocytes, MON: Monocytes, EOS: Eosinophils, BAS: Basophils, RBC: Red blood cells, HGB: Hemoglobin, HCT: Hematocrit, MCV: Mean corpuscular volume, MCH: Mean corpuscular hemoglobin, MCHC: Mean corpuscular hemoglobin concentration, PLT: Platelets.

**Table 6:** Biochemical parameters for male rats after 28 days of oral exposure to single investigated substances (DEHP, DBP, and BPA) and their mixture (MIX).

Parameter	Value Presentation	Oil Control	DEHP	DBP	BPA	MIX
Glucose (mmol/L)	Average	11.78	14.86 *	14.78 *	11.06 ^‡^	14.58 *
	SD	2.931	2.808	1.630	2.241	1.941
	% to control		26	25	−6	24
CRP (mg/L)	Average	0.4500	0.7800 *	0.7400 *	0.6000	0.7833 *
	SD	0.05477	0.1789	0.1517	0.2449	0.1169
	% to control		73	64	33	74
Urea (mmol/L)	Average	7.540	7.300	7.300	7.550	8.033 *
	SD	0.4506	0.7517	0.7517	0.7937	0.8779
	% to control		−3	−3	0	7
Creatinine (µmol/L)	Average	43	43.80	44.20	44.25	42.50
	SD	4.743	3.347	3.114	3.775	2.345
	% to control		2	3	3	−1
Uric acid (µmol/L)	Average	337.2	360.8	373.4	304.0	355.8
	SD	71.71	98.04	98.04	40.32	59.18
	% to control		7	11	−10	6
Total protein (g/L)	Average	76.20	78.80	76.40	72.50	76.17
	SD	4.207	8.408	2.074	7.506	2.639
	% to control		3	0	−5	0
Albumin (g/L)	Average	50.75	53.00	50.080	49.75	53.00
	SD	2.500	5.149	1.643	5.252	2.530
	% to control		6	1	0	6
Total bilirubin (µmol/L)	Average	1.020	1.260	1.183	1.325	1.520 *
	SD	0.3271	0.5079	0.3061	0.09574	0.1789
	% to control		24	16	30	49
Direct bilirubin (µmol/L)	Average	0.6500	0.6600	0.7600	0.8250	0.7500
	SD	0.1291	0.2966	0.1517	0.1500	0.1378
	% to control		2	17	27	15
ALT (U/L)	Average	57.8	62.8^‡^	60.0 ^‡‡^	62.8 ^‡^	73.00 **
	SD	6.140	5.933	8.396	7.463	2.309
	% to control		2	1	5	27
AST (U/L)	Average	134.6	131.4^‡^	142.0	137.8	154.5 *
	SD	7.733	8.417	12.17	18.5	12.26
	% to control		−1	7	5	15
The De Ritis ratio	Average	2.353	2.111	2.384	2.220	2.087
	SD	0.3168	0.2680	0.1841	0.4169	0.1331
	% to control		−10	1	−6	−11
ALP (U/L)	Average	363.00	422.2 *	374.7	422.5 *	385.0
	SD	36.98	57.76	12.88	38.51	34.29
	% to control		16	3	16	2
Cholesterol (mmol/L)	Average	1.584	1.274 **	1.356 *	1.796 ^‡‡‡^	1.290 *
	SD	0.2031	0.2045	0.1232	0.1623	0.1377
	% to control		0	7	18	4
HDL (mmol/L)	Average	0.9980	0.9600	0.9600	0.9400	0.8425
	SD	0.07225	0.1872	0.1581	0.1780	0.09605
	% to control		−4	−4	−6	−16
LDL (mmol/L)	Average	0.2960	0.3040	0.3680*	0.3725 *	0.3450
	SD	0.02608	0.05188	0.0497	0.05188	0.01243
	% to control		3	24	26	16.5
Triglycerides (mmol/L)	Average	1.122	1.202	1.448 * ^‡^	1.508 * ^‡^	1.115
	SD	0.1535	0.2545	0.2815	0.2973	0.1097
	% to control		7	29	34	−1
Serum iron (µmol/L)	Average	46.33	43.05	44.00	37.75 *	41.13
	SD	3.963	6.717	2.993	3.148	5.086
	% to control		−7	−5	−19	−11
Na^+^ (mmol/L)	Average	157.0	155.8	153.8	160.3	159.0
	SD	4.393	2.683	1.304	6.602	3.521
	% to control		−1	−2	1	1
K^+^ (mmol/L)	Average	8.920	9.300	9.100	8.450	8.365
	SD	1.066	1.703	0.6782	1.507	0.3061
	% to control		4	2	−5	−6
Cl^−^ (mmol/L)	Average	102.6	100.6	100.4	101.3	101.8
	SD	2.191	1.817	1.140	2.754	2.041
	% to control		−2	−2	−1	−1
Ca^2+^ (mmol/L)	Average	3.762	3.843	3.988	3.890	3.927
	SD	0.2095	0.2451	0.2895	0.1158	0.1782
	% to control		2	6	3	4
PO_4_^3−^ (mmol/L)	Average	4.910	5.520	4.954	5.410	5.227
	SD	0.3955	0.9345	0.4149	0.4859	0.4700
	% to control		12	1	10	6
Mg^2+^ (mmol/L)	Average	2.198	2.380	2.478	2.195	2.277
	SD	0.2869	0.2616	0.3268	0.2272	0.2045
	% to control		8	13	0	4

* *p* < 0.05, ** *p* < 0.01 (compared to the control), ^‡^ *p* < 0.05, ^‡‡^ *p* < 0.01, ^‡‡‡^ *p* < 0.001 (compared to the MIX group). One-way ANOVA with LSD post-hoc test/Kruskal–Wallis test with Dunn’s post-hoc test. DEHP: Bis (2-ethylhexyl) phthalate (50 mg/kg b.w.), DBP: Dibutyl phthalate (50 mg/kg b.w.), BPA: Bisphenol A (25 mg/kg b.w.), MIX: Mixture (50 mg/kg b.w. DEHP + 50 mg/kg b.w DBP + 25 mg/kg b.w BPA), CRP: C-reactive protein, AST: Aspartate aminotransferase, ALT: Alanine aminotransferase, ALP: Alkaline phosphatase, HDL: High-density lipoprotein, LDL: Low-density lipoprotein.

**Table 7:** The serum hormone level of rats orally exposed to DEHP, DBP, BPA, and their mixture (MIX) for 28 days.

Group	Value Presentation	T3 (nmol/L)	T4 (nmol/L)	T3/T4 ratio	Testosterone (ng/mL)
Oil Control		1.980 ± 0.3114	70.58 ±12.85	0.02831 ± 0.003273	3.958 ± 0.6442
DEHP		1.940 ± 0.2793	71.70 ± 13.46 ^‡^	0.02820 ± 0.008791 ^‡^	3.488 ± 1.001
	% to control	−2	1	0	−12
DBP		1.780 ± 0.1304	49.68 ± 3.975 * ^‡‡‡^	0.03608 ± 0.004508 ^‡‡‡^	2.990 ± 0.6719
	% to control	−10	−30	27	−24
BPA		1.925 ± 0.2217	53.70 ± 15.09 * ^‡‡‡^	0.03585 ± 0.01310 ^‡‡^	3.113 ± 0.6540
	% to control	−3	−24	27	−21
MIX		1.740 ± 0.3209	90.73 ± 10.56 **	0.01927 ±0.001498 *	2.680 ± 0.3492 **
	% to control	−12	28.5	−32	−32

* *p* < 0.05, ** *p* < 0.01 (compared to the control), ^‡^ *p* < 0.05, ^‡‡^ *p* < 0.01, ^‡‡‡^ *p* < 0.001 (compared to the MIX group). One-way ANOVA with LSD post-hoc test/Kruskal–Wallis test with Dunn’s post-hoc test. Values are presented as the means ± SD (*n* = 6). DEHP: Bis (2-ethylhexyl) phthalate (50 mg/kg b.w.), DBP: Dibutyl phthalate (50 mg/kg b.w.), BPA: Bisphenol A (25 mg/kg b.w.), MIX: Mixture (50 mg/kg b.w. DEHP + 50 mg/kg b.w DBP + 25 mg/kg b.w BPA), T3: triiodothyronine, T4: thyroxine.
